# Harnessing *in vitro* cytotoxicity and antibacterial potential of a novel silver-DABCO framework against multi-drug-resistant pathogens†

**DOI:** 10.1039/d5ra00509d

**Published:** 2025-03-17

**Authors:** Shivaprasad Shivappa Desai, K. A. Deepika Roy, Padikkamannil Abishad, Rahul Krishnan, Valil Kunjukunju Vinod, Asha Karthikeyan, Plantharayil Bharathan Aswathi, Sanis Juliet, Sukhadeo Baliram Barbuddhe, Deepak Bhiwa Rawool, Jess Vergis

**Affiliations:** a Department of Veterinary Public Health, College of Veterinary and Animal Sciences, Pookode, Kerala Veterinary and Animal Sciences University Wayanad 673 576 India itzjessvergis@gmail.com +91-9446355683; b Department of Aquatic Animal Health Management, Faculty of Fisheries Science, Kerala University of Fisheries and Ocean Studies Panangad Kochi 682 506 India; c Department of Poultry Science, College of Veterinary and Animal Sciences, Pookode, Kerala Veterinary and Animal Sciences University Wayanad 673 576 India; d Department of Veterinary Pharmacology and Toxicology, College of Veterinary and Animal Sciences, Pookode, Kerala Veterinary and Animal Sciences University Wayanad 673 576 India; e ICAR-National Meat Research Institute Chengicherla, Boduppal Post Hyderabad 500 092 India

## Abstract

This study aimed to synthesize and characterize silver-based metal–organic frameworks (Ag-MOFs) using 1,4-diazabicyclo[2.2.2]octane (DABCO) as the organic ligand and to assess their antibacterial and cytotoxic properties. The formation of Ag-MOF-D was confirmed by the appearance of a brown solution and a surface plasmon resonance peak at 394 nm in UV-vis spectroscopy. Fourier-transform infrared spectra showed characteristic peaks at 673, 705, 883, 1060, 1382, 1654, and 3250 cm^−1^. Powder X-ray diffraction patterns indicated a crystalline structure with peaks at 33°, 38°, 55°, and 66°, with an average particle size of 15.68 nm. Ag-MOF-D displayed thermal stability up to 650 °C with a residual mass of 91.50%. Scanning electron microscopy revealed spherical morphology with minimal aggregation, while energy-dispersive X-ray spectroscopy showed 88.64 wt% Ag^+^. Transmission electron microscopy indicated mono-dispersed spherical particles with an average diameter of 10.47 ± 1.80 nm and a lattice fringe spacing of 0.19 nm. The type II isotherm and Brunauer–Emmett–Teller analysis suggested a mesoporous structure of Ag-MOF-D with a surface area of 5.3005 m^2^ g^−1^ and an average pore diameter of 9.46 nm. Minimum inhibitory and minimum bactericidal concentration values against multi-drug-resistant bacterial strains ranged from 3.90 to 7.80 μM and 7.8 to 62.5 μM, respectively. *In vitro* cytotoxicity testing on Vero cell lines indicated a dose-dependent decrease in cell viability, with an IC_50_ value of 1.701 × 10^−2^ mg mL^−1^. These findings suggest that Ag-MOF-D holds potential for antibacterial applications and biocompatibility, with future opportunities for environmental and food safety applications.

## Abstract 2

This study aimed to synthesize and characterize silver-based metal–organic frameworks (Ag-MOFs) using 1,4-diazabicyclo[2.2.2]octane (DABCO) as the organic ligand and to assess their antibacterial and cytotoxic properties.

## Graphical abstract

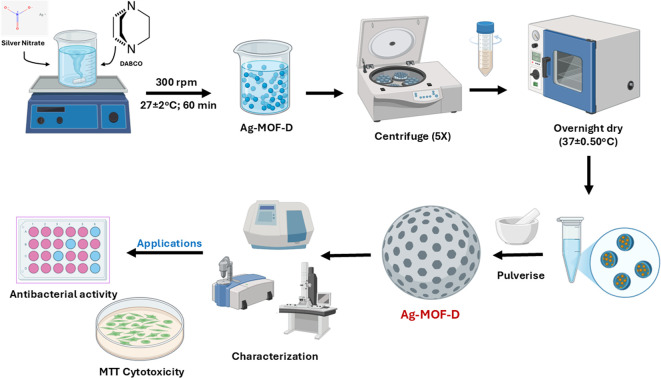

## 1 Introduction

The advent of nanotechnology and its potential have heightened optimism for further exploration in biomedical research.^1^ Nanomaterials have attracted recent biomedical attention^2^ owing to the host biocompatibility and selectivity against multi-drug-resistant (MDR) bacterial pathogens^3^ complemented with enhanced drug loading capacity, improving drug solubility and stability.^4^ Recently, better selectivity and enhanced bacterial internalization have bolstered metal–organic frameworks (MOFs) as outstanding nanomaterials for antibacterial application alternatives to conventional antibiotics to curb the crisis of antibiotic resistance.^5^ MOFs consist of metal ions or clusters connected by organic ligands, including sulfonate, carboxylate, and phosphonate functional groups. The larger and specific surface area, higher tunable porous structure, convenience in physico-chemical functional adjustability,^6^ and ability to regulate the chemical composition^7^ enable the usage of MOFs in diverse fields such as nanocarriers for targeted drug delivery,^8^ energy storage,^7^ gas separation, biomedical imaging,^9^ disease diagnosis,^10^ tissue regeneration,^11^ biological 3D printing,^12^ wastewater treatment,^13^ and dye adsorption application.^14^

Strong hydrophilicity, electronegativity,^15^ and excellent dispersibility^16^ of silver (Ag)-based nanomaterials favour its broad-spectrum antibacterial activity.^17^ The Ag^+^ ions form a coordination polymer with organic ligands^15^ resulting in a stable stereochemical crystalline structure of Ag-MOF. In addition to the applications in bioimaging and biosensors, the customizable porosity, adjustable sie, and tuneable structure of Ag-MOFs offer significant advantages over traditional drug delivery carriers like liposomes, polymers, and inorganic nanoparticles with lower drug loading capacity and instability.^18^ The biocompatibility and immune evasion properties render the utility of Ag-MOF for drug delivery and cancer therapy.^18^ The Ag-MOF structure regulates the sustained release of Ag^+^ ions, thereby ensuring biocompatibility by minimizing the cellular apoptosis caused by rapidly released Ag^+^ ions,^19^ facilitating their use in tissue engineering.^20^

Among the various established methods for synthesizing metal–organic frameworks (MOFs), including solvothermal, microwave-assisted, electrochemical, sonochemical, mechanochemical, and micro-emulsion techniques, the solution-based approach offers milder reaction conditions, enabling faster, cleaner synthesis and higher yields of nanomaterials.^21^ Of the several organic ligands used in the synthesis of MOFs, DABCO (1,4-diazabicyclo[2.2.2]octane) is a highly attractive ligand for the synthesis of MOFs due to its low cost, eco-friendliness, reactivity, ease of use, non-toxicity, and strong selectivity as a basic organocatalyst. DABCO also serves as a structural pillar in MOF synthesis, enabling the formation of stable 3D frameworks.^22^ While earlier researchers synthesized Ag-MOFs for theragnostic as well as wastewater treatment using melamine^23^ or 1,4-benzene dicarboxylate (BDC)^24^ as ligands, the DABCO-mediated Ag-MOFs appear to be scant in literature.

Recently, MOFs have garnered significant interest in nanomedicine applications, with the first such example progressing to a phase II clinical trial in humans.^25^ MOFs inherently feature a range of advantageous properties that make them highly suitable for various applications, including drug delivery, imaging, and innovative therapeutic strategies,^26,27^ often used in combination.^28^ Ag-MOFs have attracted interest in biological applications due to their antimicrobial properties, large surface area and adjustable porosity, enabling efficient drug loading and controlled release.^20^ Their luminescent features make them useful in imaging and diagnostics, while stability and chemical modifications enhance interactions with biological systems.^29,30^ Hence, this study was aimed to synthesize and characterize Ag-MOFs using DABCO as the organic ligand and evaluate their antibacterial activity against multi-drug-resistant (MDR) pathogens of public health importance. Additionally, the study aims to assess the biocompatibility of Ag-MOFs through an *in vitro* cytotoxicity assay on Vero cell lines.

## 2 Experimental

### 2.1. Bacterial isolates and chemicals

We used MDR strains of enteroaggregative *E. coli* (EAEC), *Salmonella enterica* Typhimurium, *S.* Enteritidis, and methicillin-resistant *Staphylococcus aureus* (MRSA).^31,32^

Silver nitrate (AgNO_3_, ≥99.9% purity, Sigma-Aldrich Pvt. Ltd, USA), DABCO (≥99% purity, Sigma-Aldrich, USA), and methanol (≥99.8% purity, Loba Chemie, India) were procured from commercial firms.

All the dehydrated bacterial culture media used in this study were procured from HiMedia Laboratories Pvt. Ltd. (India).

### 2.2. Synthesis of DABCO-mediated Ag-MOFs (Ag-MOF-D)

In this study, Ag-MOFs were synthesized using the organic ligand DABCO by solution method.^33^

In brief, aqueous solutions of AgNO_3_ (0.10 M; 20 mL) and DABCO (0.20 M; 80 mL) were mixed gently at 300 rpm using a magnetic stirrer (Neuation Technologies Pvt. Ltd, India) for 60 min at room temperature (27 ± 2 °C). Subsequently, the solution was allowed to settle, and the sediment was washed five times, alternatively using methanol and nanopure water. The obtained compound (Ag-MOF-D) was dried overnight in an incubator maintained at 37 ± 0.5 °C, pulverized, and stored at room temperature until further use.
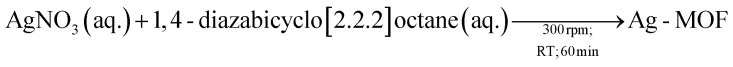


### 2.3. Characterization of Ag-MOF-D

The synthesized Ag-MOF-D was evaluated by UV-Vis spectroscopy, Fourier transform infrared spectroscopy (FTIR), powder X-ray diffraction (PXRD), thermogravimetric analysis (TGA), differential thermal analysis (DTA), scanning electron microscopy-energy dispersive X-ray analysis (SEM-EDAX), transmission electron microscopy (TEM) and Brunauer–Emmett–Teller (BET) analysis.

Initially, the Ag-MOF-D was dissolved in nanopure water to obtain a final concentration of 1 mg mL^−1^ and scanned within the range of 200 to 800 nm using a UV-Vis spectrophotometer (ThermoFisher Scientific Pvt. Ltd, USA) with a suitable blank.

The Ag-MOF-D was subjected to FTIR within a range of 400 to 4000 cm^−1^ at a resolution of 0.20 cm^−1^ (Thermo Nicolet iS50, USA) to evaluate the presence of unknown chemical functional groups.

For structural investigations of the Ag-MOF-D, PXRD (Bruker D8 Advance, USA) operated at CuKα radiation using 40 KeV and 35 mA with a scanning step size of 0.02° (*λ* = 1.54060 Å) was employed.

The thermal stability of the Ag-MOF-D was investigated in terms of TGA–DTA analysis with the aid of a simultaneous thermal analyzer (HITACHI STA7300, Japan). The analysis was done at a heating rate of 20 °C min^−1^ over 40 to 800 °C under a nitrogen atmosphere.

The morphology and elemental analysis of Ag-MOF-D were assessed by examining the samples using SEM (Jeol 6390LV, Japan) with different magnifications and EDAX, while TEM (JEM 2100, Jeol, Japan) determined its morphology and crystallinity.

The specific surface area of Ag-MOF-D was measured by low-pressure N_2_ adsorption–desorption isotherm data using an automated gas sorption analyser (BELSORP-max, Japan). The Ag-MOF-D was degassed at 100 °C for 2 h to measure N_2_ adsorption–desorption isotherms. Measurements were made in the relative pressure range (*P*/*P*_o_) of 0.04–0.4 at a temperature of 77.0 K. The specific surface area was estimated from isotherm using the BET equation,^34^ while the Barrett–Joyner–Halenda (BJH) method was used to determine the pore size.^35^

### 2.4. *In vitro* antimicrobial activity of Ag-MOF-D

The minimum inhibitory concentration (MIC) and minimum bactericidal concentration (MBC) of Ag-MOF-D, as a measure of their antibacterial efficacy, were determined^31^ against MDR strains of EAEC, *S.* Typhimurium, *S.* Enteritidis, and MRSA.

In brief, the individual bacterial cultures (100 μL; *ca.* 1 × 10^7^ CFU mL^−1^) grown on sterile cation-adjusted Mueller–Hinton broth were co-incubated with decreasing concentrations of Ag-MOF-D (1000 to 0.24 μM), keeping appropriate controls in a 96-well microtitre plate. The untreated bacterial culture and sterile media served as positive and negative controls, respectively. The microtiter plate was incubated at 37 ± 0.5 °C for 18–24 h. Then, resazurin dye (20 μL; 0.015% w/v) was added to the wells to determine the colour change, indicating the degree of bacterial inhibition.

The MIC value was determined as the least concentration of Ag-MOF-D without exhibiting visible growth, whereas the MBC was determined using the inoculum (10 μL) drawn from those wells displaying no visible bacterial growth and plating on selective agar plates.^36^ In this study, Eosin-Methylene Blue, Xylose Lysine Deoxycholate, and Baird-Parker agar plates were used for selective plating of EAEC, *Salmonella* spp., and MRSA, respectively. The MBC of Ag-MOF-D was estimated to be the lowest concentration required to kill bacterial strains on selective agar by 99.90%.^37^

### 2.5. *In vitro* viability assay on Vero cell lines

The effect of Ag-MOF-D on the survival of Vero cells was assessed *in vitro* through the MTT assay.^38^ The Vero cells used in this assay were procured from the National Centre for Cell Science (NCCS), Pune.

Briefly, the Vero cells were pre-cultured in tissue culture flasks containing Dulbecco's Modified Eagle Medium (DMEM) supplemented with 10% fetal bovine serum (FBS; Gibco, USA) until the formation of a monolayer at the bottom of the flasks. The adherent cells were then transferred to another 96-well plate at a density of 1 × 10^4^ cells per well and allowed to attach overnight. The monolayers of cells were treated with 100 μL of Ag-MOF-D at different concentrations (10^−1^ to 10^−9^ mg mL^−1^) diluted in DMEM starting from an initial 1000 mg mL^−1^ concentration, while the cells incubated with sterile DMEM served as the negative control. The treated cells were maintained for 24 h at 37 ± 0.50 °C in a humidified incubator with a 5% CO_2_ atmosphere.

The supernatant was removed, proceeded further using the MTT cell proliferation assay kit (Sigma-Aldrich, USA) and the cytotoxicity was monitored by measuring the absorbance at 550 nm and estimated as,Cytotoxicity (%) = 100 × (control − sample)/(control)wherein, the control denotes the experimental absorbance of the untreated cell control, and the sample represents the control absorbance of the treated cell lines.

The half-maximal inhibitory concentration (IC_50_) was estimated to evaluate the cytotoxic effect of Ag-MOF-D synthesized by the solution method in Vero cells.

### 2.6. Statistical analysis

All experiments were performed three independent times in triplicates. The data obtained were represented as means ± standard deviations analyzed using GraphPad Prism version 8.4.2 (GraphPad Software Inc., California, USA). A non-linear regression (dose–response) was used to estimate the IC_50_ of Ag-MOF-D, while one-way analysis of variance (ANOVA) with Bonferroni multiple comparison post-test was used to compare the differences between the cytotoxicity of control and Ag-MOF-D-treated cell lines. A *P*-value ≤ 0.01 was considered highly significant, while a *P*-value ≤ 0.05 was considered statistically significant.

## 3 Results and discussion

### 3.1. Synthesis of Ag-MOF-D

**Fig. 1:**
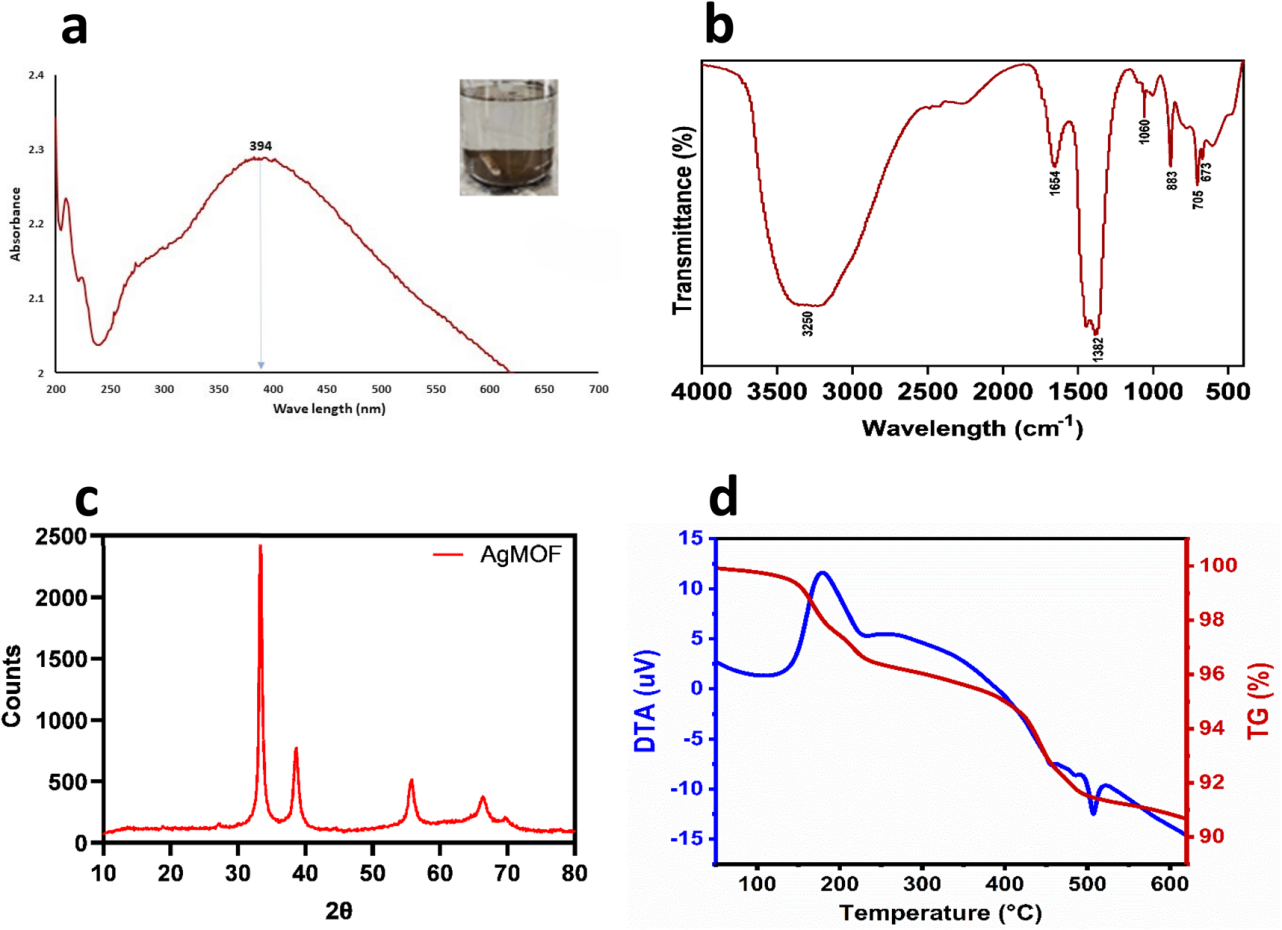
Physicochemical characterisation of Ag-MOF-D. Images (a) denotes UV-vis spectrum, (b) represents FTIR spectrum, (c) demonstrates XRD pattern, (d) denotes TGA–DTA plot.

MOFs are typically crystalline 3D coordination polymers created by hybridizing metal ions or clusters with organic ligands or polymers/linkers through coordination bonds.^39^ In this study, AgNO_3_ (substrate for Ag) reacted with DABCO (organic ligand), reducing Ag^+^ ions. The reduction of metal ions triggers the excitation of surface plasmon resonance (SPR),^40^ indicating the formation of Ag-MOF-D evidenced by the appearance of a brown-coloured solution (Fig. 1a).

The ease of structural customization and versatility during the synthesis of MOFs have made the use of DABCO a more feasible organic ligand. This appears to be the first study of its kind in utilizing DABCO as an organic ligand to synthesize Ag-MOF. Incorporating DABCO has been hypothesized to improve the construction of MOFs with antibacterial properties and enhanced biocompatibility for versatile biological applications.^24,41^ Moreover, DABCO has widely been used in a variety of reactions since it is eco-friendly, reactive, and non-toxic with a considerable degree of selectivity.^22^ Hence, this study employed DABCO as an organic ligand for synthesizing Ag-MOF.

### 3.2. Structural and morphological characterization of Ag-MOF-D

Initially, the prominent SPR peak observed at 394 nm by UV-vis spectroscopy (Fig. 1a) confirmed the synthesis of Ag-MOF-D.^42^ The broader base observed at the absorption peak could indicate agglomerated AgNPs. This agglomeration likely results in strong Coulomb repulsion among AgNPs, which can help stabilise the AgNPs despite their clustering.^43^

The FTIR spectra of the synthesized Ag-MOF-D exhibited characteristic peaks at 673, 705, 883, 1060, 1382, 1654, and 3250 cm^−1^ (Fig. 1b). The peak observed at 673 cm^−1^ corresponds to C–H deformation in the aromatic ring at the alkene C–H bond,^44^ while the peak at 705 cm^−1^ indicates the confined amine chains^45^ in the organic ligand. The FTIR peak at 883 cm^−1^ suggests that transition metal (Ag) and ligand (DABCO) combination safe been widely recognized as highly effective catalytic systems for C–C bond formation.^22^ The band observed at 1060 cm^−1^ in the FTIR spectrum is indeed attributed to complex molecular vibrations, primarily involving the in-phase antisymmetric stretching vibrations of the NC3 groups (in-ν_a_(NC_3_)) of DABCO (1,4-diazabicyclo[2.2.2]octane), the antisymmetric stretching vibrations of C–C bonds (ν_a_(C–C)), and the twisting vibrations of the methylene groups (γ_t_(CH_2_)).^46^ The strong peak at 1382 cm^−1^ likely corresponds to C–N stretch vibrations in the DABCO,^47^ while the band at 1654 cm^−1^ may indicate C=N stretching vibrations.^48^ The band at 3250 cm^−1^ corresponds to O–H stretching vibrations of the DABCO.^49^

The PXRD peaks (2*θ*) of Ag-MOF-D were observed at 33°, 38°, 55°, and 66° (Fig. 1c). The broad Bragg's diffraction peak in XRD would be attributed to the crystallite size and lattice strain.^50^ The intensity of PXRD peaks reflected the degree of crystallinity of the Ag-MOF-D. Moreover, the broader peaks observed in the XRD (Fig. 1c) would correlate with the smaller crystalline size, as they are inversely related to each other.^50^ In addition, the particle size, as determined by PXRD, has an average of 15.68 nm by Debye–Scherrer's equation.^23^

The TGA–DTA was performed to analyse the structural stability and thermal degradation of Ag-MOF-D (Fig. 1d–f). In this study, the Ag-MOF-D exhibited two distinct weight loss phases: the first phase, with a 1.50% weight loss, was observed between 148.7 and 175 °C, retaining approximately 98.50% of the structural stability. This minimal decomposition could likely be due to removing residual solvents and organic compounds in Ag-MOF-D.^51^ The second phase, with a weight loss of 7% observed between 421.4 and 453 °C, retained about 93% of its structural stability, which is attributed to the decomposition of DABCO and the structural collapse of the framework.^52^ Furthermore, the DTA analysis revealed a gradual thermal degradation between 180 and 235 °C, along with an exothermic peak at 179 °C which may be due to decomposition or structural transition of the Ag-MOF-D, similar to previous observations^53^ another endothermic peak observed at 506.9 °C due to melting or phase transition. In addition, the Ag-MOF-D remained thermally stable up to 650 °C, exhibiting a total residual mass of 91.50%. The organic ligand employed in this study, DABCO, is a nitrogen-containing heterocyclic compound that coordinates with Ag^+^ to form robust bonds, incorporating high-energy functional groups such as –NO_2_, –ONO_2_, and –N_3_. Additionally, the three-dimensional MOF structure formed with DABCO likely contributes to enhanced structural stability, exhibiting reduced thermal sensitivity.^22,41^

**Fig. 2:**
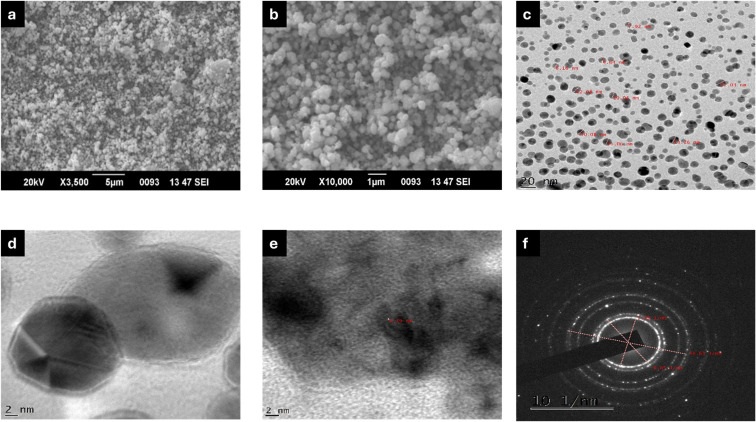
Electron micrographs of Ag-MOF-D. Images (a and b) represents SEM micrographs, (c and d) demonstrates TEM micrographs, (e) exhibits lattice fringing, and (f) illustrates SAED pattern.

The SEM micrographs of Ag-MOF-D demonstrated the spherical surface morphology with minimal aggregation (Fig. 2a and b). In this study, a single SPR peak of Ag-MOF-D noticed at 394 nm indicated AgNPs with a spherical morphology.^54^ Moreover, EDAX analysis indicated a high intensity of Ag^+^ concentration, *i.e.*, 88.64 wt% (ESI Fig. 1†). This significant concentration of Ag^+^ on the surface of Ag-MOF-D contributes to its enhanced antibacterial activity. However, the presence of O in EDAX data might have originated from the biomolecules bound to the surface of AgNPs, indicating the reduction of Ag^+^ ions into elemental Ag.^55^ The presence of Si in trace amounts could be due to the introduction of impurities during the synthesis of nanomaterials.^56^ In addition, TEM analysis revealed a mono-dispersed spherical morphology, with an average diameter of 10.47 ± 1.80 nm (Fig. 2c and d) and a lattice fringe spacing of 0.19 nm (Fig. 2f). The SAED patterns (Fig. 2g), along with XRD data, confirmed the crystalline nature of Ag-MOF-D.

### 3.3. Specific surface area and pore size distribution of Ag-MOF-D

**Fig. 3:**
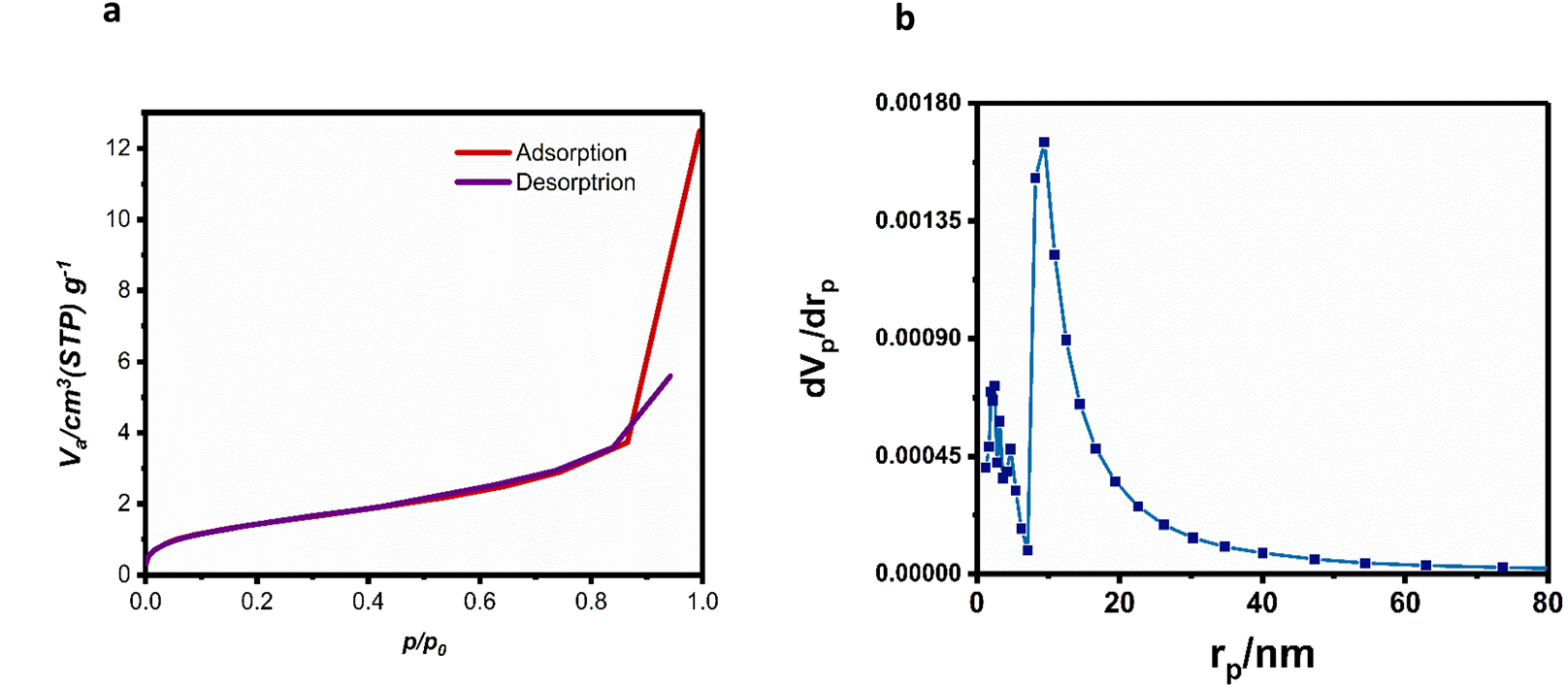
Specific surface area and pore size distribution of Ag-MOF-D. Images (a) represents BET plot and (b) denotes BJH plot.

The specific surface area and porosity are crucial parameters for characterizing MOFs. The irregular surface morphology of these materials enhances gas adsorption by facilitating a pore-filling mechanism during BET analysis.^57^ The type II isotherm curve of Ag-MOF-D (Fig. 3a), as classified by the International Union of Pure and Applied Chemistry, suggested that Ag-MOF-D possesses a mesoporous structure. The gradual curvature (Fig. 3a) suggests a substantial overlap of monolayer coverage, marking the onset of multilayer adsorption.^58^ The adsorption of N_2_ occurred over relative pressures of 0.04–0.99 cm^3^ g^−1^, reaching the maximal level of 12.486 cm^3^ g^−1^.

In contrast, the desorption occurred over 0.942–0.001 cm^3^ g^−1^ with a minimal level of 0.3174 cm^3^ g^−1^. The BET analysis (ESI Fig. 2†) evaluated the porous nature of Ag-MOF-D with a surface area measurement of 5.3005 m^2^ g^−1^. The BJH analysis results (Fig. 3c) revealed that the average pore diameter of the material is 9.46 nm. The pore size distribution, ranging from 9 to 25 nm, indicates that Ag-MOF-D has a relatively uniform pore structure. The small size of MOFs contributes to their large surface area, which enhances the interaction between the MOF material and bacterial cells. This increased surface interaction facilitates better penetration of the MOFs into bacterial cell membranes.^16^ MOFs are considered as a good porous material that provides a reservoir to metal ions and controls their sustained release, enabling antibacterial activity.^59^

### 3.4. *In vitro* antimicrobial activity of Ag-MOF-D

The antibacterial activity of Ag-MOF-D was evaluated by determining the MIC as well as MBC values against MDR strains of *S.* Typhimurium, *S.* Enteritidis, EAEC, and MRSA (Table 1). In this study, the MIC of Ag-MOF-D varied within 3.90–7.80 μM, whereas the MBC values ranged from 7.8 to 62.5 μM, with strain variation (Table 1).

**Table 1:** *In vitro* antimicrobial activity of Ag-MOF-D

MDR-isolates	Isolate ID	MIC (μg mL^−1^)	MBC (μg mL^−1^)
EAEC	E1	3.9	7.8
	E2	3.9	7.8
	E3	3.9	7.8
*S.* Typhimurium	ST1	7.8	62.5
	ST2	7.8	31.2
	ST3	7.8	31.2
*S.* Enteritidis	SE1	7.8	31.2
	SE2	7.8	31.2
	SE3	7.8	31.2
MRSA	SA1	3.9	15.6
	SA2	3.9	7.8
	SA3	3.9	15.6

The antibacterial efficacy of Ag-MOF-D lies in the liberation of Ag ions;^15^ factors such as the shape, chemical composition, and the framework with coordination ligands play significant roles in determining the rate and amount of the release. Additionally, the porosity and the nature of ligands can further affect the kinetics of metal ion diffusion, influencing the material's overall antimicrobial activity. Moreover, Ag-MOFs enter bacterial surfaces, releasing Ag^+^ ions that disturb ion balance, impair ion channels, interact with membrane constituents (lipophilic acids, hydroxyl groups in peptidoglycan, and phosphate groups in phospholipids), and attach to thiol groups in proteins. This process deactivates key metabolic enzymes and undermines the integrity and permeability of bacterial membrane.^60^ In addition, the reactive oxygen species (ROS) generated upon induction by Ag^+^ ions would inhibit bacterial nucleic acid damage.^61^

Interestingly, the EAEC and MRSA strains exhibited comparatively lower MIC and MBC values than the *Salmonella* spp. tested in this assay.

### 3.5. *In vitro* cell viability assay in Vero cells

**Fig. 4:**
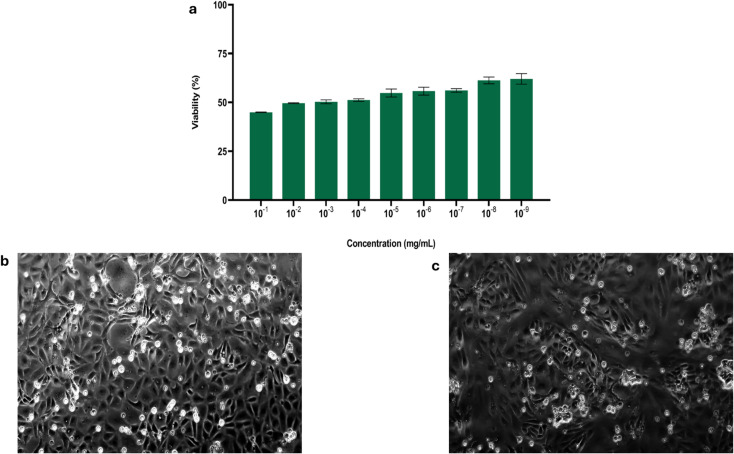
*In vitro* cell viability assay in Vero cells. Images (a) demonstrates *in vitro* viability of Vero cells treated with Ag-MOF-D, (b) exhibits microscopic image of untreated Vero cells and (c) represent microscopic image of Vero cells treated with Ag-MOF-D.

An *in vitro* cell viability assay was conducted to determine the safety of Ag-MOF-D in eukaryotic Vero cell lines. In this study, a dose-dependent viability of Vero cells was observed with increasing concentrations of Ag-MOF-D (10^−1^ to 10^−9^ mg mL^−1^; Fig. 4a). Nevertheless, the viability did not tend to be lower than 44.90% even at highest tested concentration (10^−1^ mg mL^−1^) of Ag-MOF-D. Moreover, this study exhibited mild to moderate cytopathic effects in the Vero cells, including loss of monolayer and syncytia formation on treatment with Ag-MOF-D (Fig. 4c) against the Vero cell control (Fig. 4b). In addition, an IC_50_ value of 1.701 × 10^−2^ mg mL^−1^ was determined for Ag-MOF-D using a non-linear regression method.

The cytotoxic properties of Ag-based MOFs hinge primarily on their ability to infiltrate cells through mechanisms such as diffusion, translocation, or phagocytosis.^62,63^ However, the ligands used often decide the cytotoxicity by modulating the release of Ag^+^ ions.^60,64^ The use of DABCO as a pillar in the synthesis of Ag-MOF-D in this assay might have reduced the cytotoxicity by controlling the release of Ag^+^ ions.^22,24,41^ The moderate cytopathic effects exhibited on treatment with Ag-MOF-D could be attributed to its capacity to penetrate membranes of Vero cells *via* diffusion and phagocytosis, in conjunction with its generation of reactive oxygen species.^61^ Meanwhile, the antibacterial activity of pure Ag-MOFs mainly stems from the release of Ag^+^ ions. This release, facilitated by weakened ligand–metal bonds or cooperative effects, disrupts bacterial cell membranes, generates ROS, and intercalates DNA, ultimately killing bacteria. MOFs generally show lower cytotoxicity compared to other nanosystems, closely linked to their composition.^19^

Previous studies commonly used benzene-1,3,5-tricarboxylic acid (BTC),^65^ benzenedicarboxylic acid (BDC),^66–68^ naphthalene-2,6-dicarboxylic acid (NDC), and [1,1′:4′,1′′-terphenyl]-4,4′′-dicarboxylic acid (TDC)^69^ to synthesize Ag-based MOFs, which demonstrated potential as effective, long-term antibacterial agents, with high stability under physiological conditions. In contrast, this study uses DABCO as an organic ligand for Ag-MOFs, valued for its low cost, eco-friendliness, non-toxicity, reactivity, and role in forming stable 3D frameworks. While this study provides valuable insights into the properties and antibacterial potential of Ag-MOF-D, several key areas would benefit from further investigation before its potential translation to biomedical applications. Future studies should incorporate zeta potential analysis and dynamic light scattering to more comprehensively assess colloidal dispersion stability and hydrodynamic diameter. Additionally, further structural analysis of Ag-MOF would provide a deeper understanding of its properties. Expanding antibacterial testing to include a broader range of bacterial strains, as well as conducting safety, stability, and *in vivo* assays, will be crucial for fully evaluating its therapeutic potential. These steps will help refine the understanding of Ag-MOF-D efficacy and pave the way for its potential biomedical applications.

## 4 Conclusion

We successfully synthesized three-dimensional Ag-MOF using a solution approach that involved DABCO as the organic ligand for the first time. The synthesis was assessed through physico-chemical assays, revealing that the crystalline Ag-MOF-D exhibited a spherical morphology and excellent thermal stability. The ability to control the sustained release of Ag^+^ ions enhanced the notable antibacterial properties of Ag-MOF-D, resulting in a low MIC and effective antibacterial activity against the test MDR strains of EAEC, *Salmonella* spp., and MRSA. In addition, Ag-MOF-D has demonstrated biocompatibility *in vitro*, as shown in studies using Vero cell lines. Optimizing the molecular building blocks and fine-tuning the size and shape of Ag-MOFs holds significant promise for enhancing their antibacterial effectiveness. Developing these Ag-MOF-based materials can pave the way for innovative applications in environmental remediation, food packaging, and antibacterial coatings. Such advancements are expected to contribute substantially to public health and safety, offering effective solutions to mitigate bacterial contamination across various sectors.

## Data availability

The data supporting this article have been included as part of the ESI.† Further, data will be made available upon request.

## Author contributions

The study was conceived and designed by [Sukhadeo Baliram Barbuddhe, Deepak Bhiwa Rawool and Jess Vergis]. Material preparation, data collection and analysis were performed by [Shivaprasad Shivappa Desai, K. A. Deepika Roy, Padikkamannil Abishad and Rahul Krishnan]. Data curation and formal analysis was performed by [Valil Kunjukunju Vinod, Asha Karthikeyan, Plantharayil Bharathan Aswathi, Sanis Juliet]. The first draft of the manuscript was written by [Shivaprasad Shivappa Desai, K. A. Deepika Roy, Padikkamannil Abishad], all authors commented on previous versions of the manuscript and final editing was performed by [Jess Vergis, Sukhadeo Baliram Barbuddhe and Deepak B. Rawool]. The work was supervised by [Jess Vergis]. All authors read and approved the final manuscript.

## Conflicts of interest

There are no conflicts to declare.

## Supplementary Material

RA-015-D5RA00509D-s001
