# Supplementary figures and images for: Harnessing in vitro cytotoxicity and antibacterial potential of a novel silver-DABCO framework against multi-drug-resistant pathogens
Source: RSC Adv. 2025 Mar 17;15(11):8180–8. doi: 10.1039/d5ra00509d (PMC11912354; doi:10.1039/d5ra00509d)

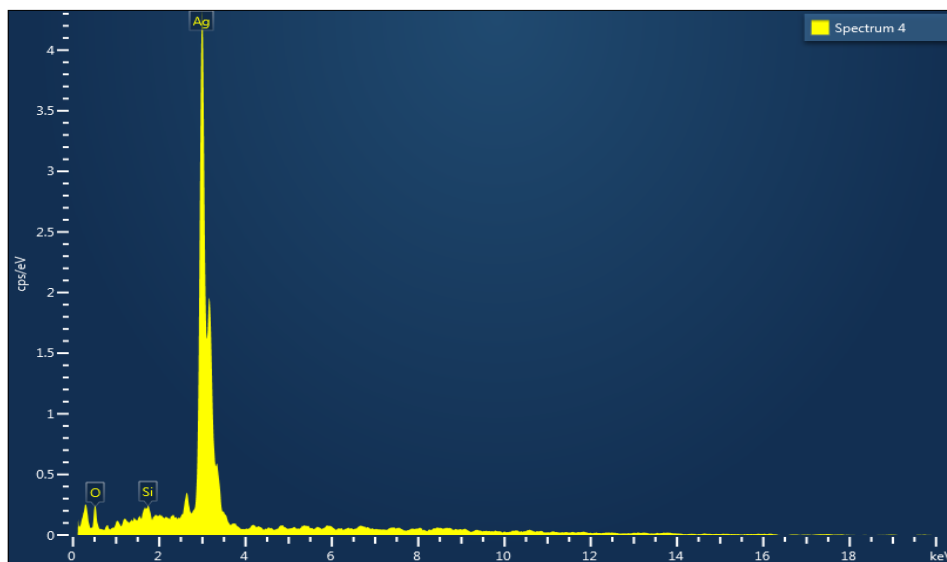

Supplementary Fig. 1. EDAX data of Ag-MOF-D

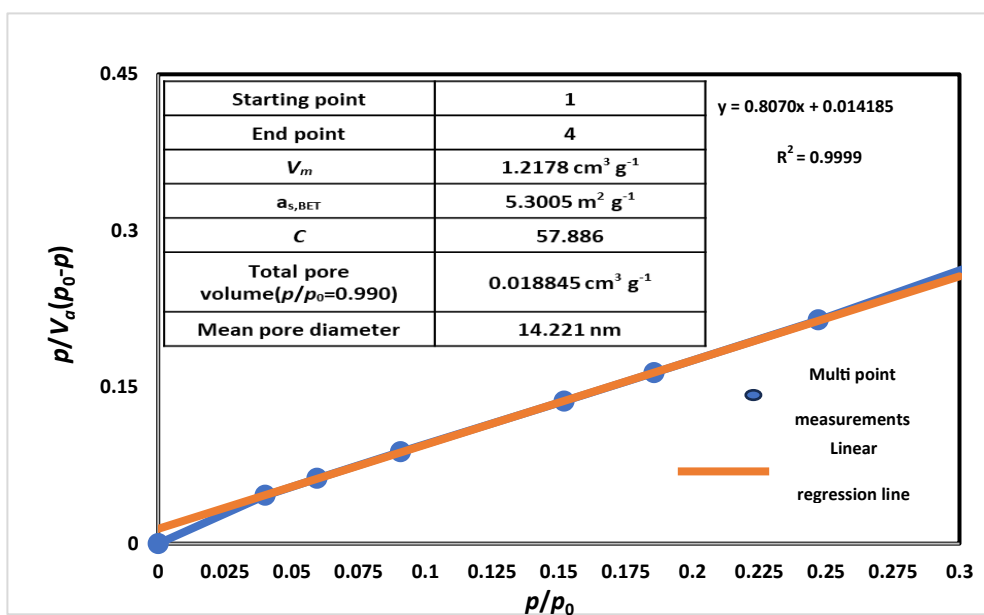

Supplementary Fig. 2 BET analysis plot

Supplement: RA-015-D5RA00509D-s001 [file RA-015-D5RA00509D-s001.pdf]
